# Hole-Transporting Low-Dimensional Perovskite for Enhancing Photovoltaic Performance

**DOI:** 10.34133/2021/9797053

**Published:** 2021-05-28

**Authors:** Fangfang Wang, Qing Chang, Yikai Yun, Sizhou Liu, You Liu, Jungan Wang, Yinyu Fang, Zhengchun Cheng, Shanglei Feng, Lifeng Yang, Yingguo Yang, Wei Huang, Tianshi Qin

**Affiliations:** ^1^Key Laboratory of Flexible Electronics (KLOFE) & Institute of Advanced Materials (IAM), Nanjing Tech University (NanjingTech), 30 South Puzhu Road, Nanjing 211816, China; ^2^Shanghai Synchrotron Radiation Facility (SSRF), Shanghai Advanced Research Institute, Shanghai Institute of Applied Physics, Chinese Academy of Sciences, 239 Zhangheng Road, Shanghai 201204, China; ^3^State Key Laboratory of Organic Electronics and Information Displays & Institute of Advanced Materials (IAM), Nanjing University of Posts & Telecommunications, 9 Wenyuan Road, Nanjing 210023, China; ^4^Frontiers Science Center for Flexible Electronics (FSCFE) & Shaanxi Institute of Flexible Electronics (SIFE), Northwestern Polytechnical University (NPU), 127 West Youyi Road, Xi'an 710072, China; ^5^Ningbo Institute of Northwestern Polytechnical University, 818 Qingyi Road, Ningbo 315103, China

## Abstract

Halide perovskites with low-dimensionalities (2D or quasi-2D) have demonstrated outstanding stabilities compared to their 3D counterparts. Nevertheless, poor charge-transporting abilities of organic components in 2D perovskites lead to relatively low power conversion efficiency (PCE) and thus limit their applications in photovoltaics. Here, we report a novel hole-transporting low-dimensional (HT2D) perovskite, which can form a hole-transporting channel on the top surface of 3D perovskite due to self-assembly effects of metal halide frameworks. This HT2D perovskite can significantly reduce interface trap densities and enhance hole-extracting abilities of a heterojunction region between the 3D perovskite and hole-transporting layer. Furthermore, the posttreatment by HT2D can also reduce the crystal defects of perovskite and improve film morphology. As a result, perovskite solar cells (PSCs) can effectively suppress nonradiative recombination, leading to an increasement on photovoltage to >1.20 V and thus achieving >20% power conversion efficiency and >500 h continuous illumination stability. This work provides a pathway to overcome charge-transporting limitations in low-dimensional perovskites and delivers significant enhancements on performance of PSCs.

## 1. Introduction

Metal-halide perovskites have been tremendously developed over the past several years because they can offer the promise of easy fabrication, low-cost solution processability, flexible substrate compatibility, broad bandgap tunability, and integration possibility of tandem multijunction architecture [1–5]. Owing to the excellent intrinsic properties of perovskite materials, such as extremely high absorption coefficient and ultralong charge carrier diffusion distance, given by the unique three-dimensional (3D) ABX_3_ framework of perovskite polycrystals [6, 7], perovskite solar cells (PSCs) have achieved very impressive power conversion efficiencies (PCEs) already exceeding 25% [8]. Despite this remarkable achievement, however, the unacceptable vulnerability of 3D perovskites to humidity and ambient atmosphere rises to a barrier toward their market uptake. In overcoming the drawbacks, 2D and quasi-2D perovskites formed by inserting bulky organic spacer cations, which cannot fit into the octahedral network but can effectively passivate interfacial defects and vacancies outside ABX_3_ frameworks, have been considered as promising materials to solve the stability issue of PSCs [9–12].

2D and quasi-2D organometallic halide perovskites are formed by splitting along lattice orientations (010) and (110) from 3D perovskites, which are identified as Ruddlesden–Popper (RP) perovskites [13, 14]. These RP layered phase perovskites consist of two components: (i) layered organic phase including large molecular cations (e.g., phenylethyl ammonium (PEA) [15, 16], butylammonium (BA) [17, 18], and 1-naphthylmethylammonium (NMA)) [19] and (ii) bulky perovskite phase containing small cations (e.g., cesium (Cs), methylammonium (MA), and formamidinium (FA)) into metal halide [BX_6_]^4−^ octahedral units [20, 21]. Recently, the 2D/quasi-2D halide perovskites define a promising and efficient emerging materials for solid-state photovoltaics [22–25]. However, it remains a great challenge to further advance the photovoltaic efficiencies. The relatively poor PCEs of 2D/quasi-2D PSCs are attributed to the suppression of out-of-plane charge transport by the organic cations, which acted like insulating spacers between the conducting inorganic slabs [26]. In this regard, large organic cations with outstanding charge-carrier mobilities might provide a promising way to solve the efficiency issues of 2D/quasi-2D PSCs. So far, the purposeful design on hole-transporting 2D perovskite in PSCs has not yet been reported.

**Figure 1:**
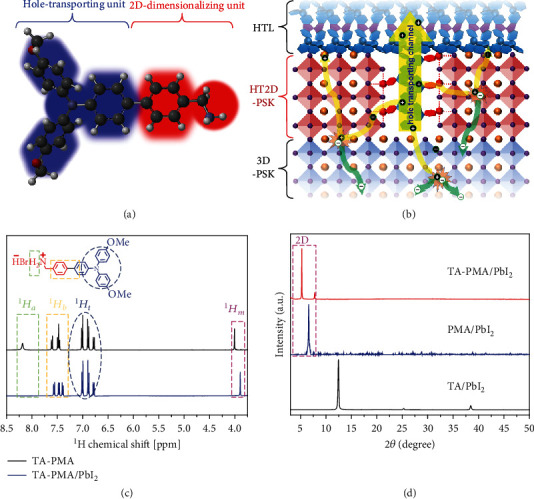
Structural analysis of TA-PMA and HT2D: (a) chemical structure of TA-PMA including a hole-transporting unit and 2D-dimensional unit; (b) illustrative scheme: TA-PMA intercalation induced formation of HT2D perovskite capping layer on 3D perovskite; (c) ^1^HNMR spectra of TA-PMA and TA-PMA/PbI_2_. The inset is the molecule structure of TA-PMA; (d) XRD patterns of TA-PMA/PbI_2_, PMA/PbI_2_, and TA/PbI_2_.

Herein, we reported a novel design strategy toward an efficient bifunctional organic salt (TA-PMA) consisting of two subunits: (i) a phenylmethylammonium (PMA) cation group (red part in Figure 1(a)) for building up RP layered phase perovskites and (ii) a triarylamine (TA) group (blue part in Figure 1(a)) for efficient hole extraction from RP perovskite to the hole-transporting layer (HTL). This tailored TA-PMA could induce a surface-2D/bulk-3D hierarchy perovskite structure, which not only can improve the stability of PSCs against damp environment due to the hydrophobic nature of formed RP perovskite but also is expected to enable a hole-transporting channel between RP perovskite slabs because of the hole-extracting ability of the TA functional group (Figure 1(b)). Moreover, the BA cation could naturally provide the desired passivation effect by forming an adduct with uncoordinated halide ions [27]. In addition, HT2D posttreatment can eliminate the miscellaneous phase of perovskite and thus improve the film morphology. Benefiting from these merits, the n-i-p planar PSC achieved a champion power conversion efficiency (PCE) of 20.71% as well as excellent operational stability exhibiting 92% of initial efficiency after 500 h continuous illumination without encapsulation.

## 2. Results

### 2.1. Characterization of TA-PMA

The TA-PMA was synthesized from a straightforward route with simple purifications and high yields (Figure S1). The chemical structures of target TA-PMA was characterized by ^1^*H*-NMR (Figure 1(c)), presenting hydrogen signals of the ammonium group (^1^*H*_a_) at 8.186 (s, 3H) ppm, the benzyl group closer to the ammonium (^1^*H*_b_) at 7.616 (d, 2H) and 7.496-7.454 (m, 4H) ppm, and the triarylamine group (^1^*H*_t_) at 7.022 (d, 4H), 6.902 (d, 4H), and 6.792 (d, 2H) ppm, as well as the methylene group (^1^*H*_m_) at 4.009 (s, 2H) ppm, respectively, in a stoichiometric ratio. The entire ^1^*H*-NMR spectra of reaction intermediate and the product are shown in Figure S2 and Figure S3. In order to identify the interaction of TA-PMA and lead iodide (PbI_2_), we mixed them at a molar ratio of 2 : 1, then observed that ^1^*H*_a_ disappeared, and ^1^*H*_b_ shifted to 7.574 (d, 2H), 7.479 (d, 2H), and 7.404 (d, 2H) ppm, (^1^*H*_m_) shifted to 3.895 (s, 2H) ppm, but ^1^*H*_t_ was almost unchanged. These chemical shifts were attributed to varying distances between protonated ammonium and neighboring hydrogens, indicating that the reaction of TA-PMA and PbI_2_ mainly occurred by the amine group and could potentially achieve a 2D perovskite (*n* = 1) phases at a molecular level. Moreover, to observe these 2D layered structures in solid state, X-ray diffraction (XRD) patterns of TA-PMA doped with PbI_2_ at a ratio of 2 : 1 (mol) were measured using phenylmethylammonium (PMA) bromide and dimethoxy-triarylamine (TA) as references under the same condition (Figure 1(d)). Only TA-PMA/PbI_2_ and PMA/PbI_2_ mixture films demonstrated significantly RP layered 2D peaks at 5.3° and 6.6° (marked in Figure 1(d)), respectively. The diffraction angle of PMA-treated 2D perovskite was similar to previous literature [28], whereas the smaller diffraction angle of TA-PMA-treated perovskite was following the tendency of its larger *d*-spacing distance owing to a bulkier structure than that of PMA [29]. In contrast, TA/PbI_2_ films displayed no extra peaks due to the lack of a reactive cation group.

### 2.2. First-Principle Calculations of TA-PMA/Perovskite Interface

**Figure 2:**
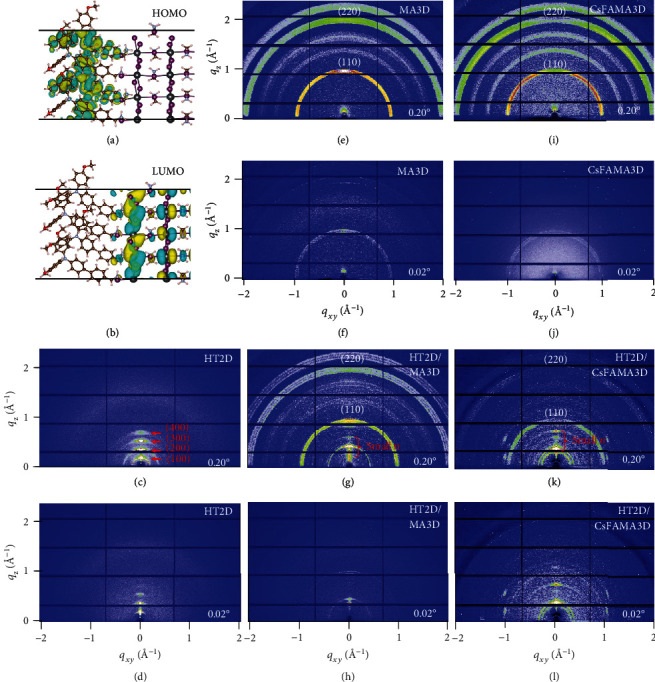
Structure characterization of HT2D/3D hierarchy perovskite: (a, b) DFT-optimized geometries of TA-PMA/ABX_3_ perovskite interface and electron density plots of HOMO (a) and LUMO (b); (c, d) 2D-GIWAXS patterns of neat HT2D in the bulk phase (c) and top surface (d) with incident angle at 0.20° and 0.02°; (e–h) 2D-GIWAXS patterns of MA3D (e, f) and HT2D/MA3D (g, h) MAPbI_3_ films at 0.20° and 0.02°; (i–l) 2D-GIWAXS patterns of CsFAMA3D (i, j) and HT2D/CsFAMA3D (k, l) films at 0.20° and 0.02°.

To shed light into the interface between TA-PMA and perovskite, we performed the density functional theory (DFT) calculations. The PMA cation unit in TA-PMA could fully embed into the A-site at the (010) lattice plane of ABX_3_ 3D perovskite, even assuming maximum 100% coverage (see the CIF file and computational details in Supporting information). The pendant TA units displayed a highly orientated arrangement along with the octahedral [PbI_6_]^4−^ framework with a spatial distance at ~17 Å. In order to further understand the TA-PMA orientation and packing behaviors at the MAPbI_3_ interface, we compared N-N atom distances at different subunits of PMA (~6.302 Å), TA (~6.401 Å), and MA (~6.435 Å) in the DFT file, respectively. These similar N-N distances could reveal a matchable interlocked structure between TA-PMA cations and MAPbI_3_ framework, as shown in Figure S4. Moreover, for further understanding, the interfacial charge transportation mechanism was illustrated. It is observed that the partial charge densities of the highest occupied molecular orbital (HOMO) are mainly located at the domain of TA-PMA slabs (Figure 2(a)), whereas those of the lowest unoccupied molecular orbital (LUMO) are distributed in the perovskite region (Figure 2(b)). These obvious distinctions between HOMO and LUMO charges indicates that the hole transportation direction at the interface is from perovskite to TA-PMA. Density of state is also calculated to illustrate the electronic change (Figure S5). The relative energy difference of TA-PMA and MAPbI_3_ is ~1.7 eV, which is identical to the experimental energy gap between HOMO of TA-PMA (−5.6 eV) and LUMO of MAPbI_3_ (−3.9 eV) [30]. Herein, we named this novel structure consisting of TA-PMA and perovskite as a hole-transporting 2D perovskite (HT2D). In other words, the spontaneously grown HT2D perovskite can make a contribution to the hole extraction from the perovskite photoactive layer to the hole-transporting layer (HTL), thus restricting the interfacial charge recombination.

### 2.3. Structure Characterization of HT2D/3D Hierarchy Perovskite

To investigate the hierarchy structure of HT2D/3D perovskite films, the synchrotron-based grazing-incident wide-angle X-ray scattering (GIWAXS) was conducted on a series of samples, including neat HT2D perovskite, pristine 3D MAPbI_3_ (3D), and hierarchy HT2D/3D perovskite based on TA-PMA posttreatment (see experimental details in Supporting information). GIWAXS measurements were performed as a function of the X-ray incident angle (*α*), providing depth-sensitive information in 2D-scattering patterns. X-ray beam could fully penetrate the hierarchy film samples with *α* = 0.20°, whereas it only analyzes the top surface if under critical angle *α* = 0.02°. In Figures 2(c) and 2(d), a neat HT2D sample prepared by stoichiometrical TA-PMA and PbI_2_ exhibited scattering patterns at *qz* ~ 0.19, 0.38, 0.57, and 0.76 Å^−1^, corresponding to (100), (200), (300), and (400) crystal faces of a RP layered 2D (*n* = 1) perovskite, respectively. These diffraction data resulted in the lamellar stacking distance between perovskite octahedral units which was ca. 33 Å, which was in coincidence with simulated double RP layered distance as first-principle calculation results. Herein, the above HT2D (*n* = 1) can be formed by the following reaction):

(1) $$\begin{array}{c} \text{Pb}{\text{I}}_{2}+2\left(\text{TA}-\text{PMA}\right)\text{Br}⟶{\left(\text{TA}-\text{PMA}\right)}_{2}\text{Pb}{\text{I}}_{2}{\text{Br}}_{4} \end{array}$$

In Figures 2(e) and 2(f), the MAPbI_3_ film demonstrated uniform 3D perovskite (110) and (220) diffraction rings in both surface and bulk phases. To form the HT2D/3D structure, TA-PMA in isopropanol (IPA) solution was spin-coated on the MA3D film and then, the posttreated film was annealed for 10 min. Figures 2(g) and 2(h) presented a direct evidence on this hierarchy HT2D/3D structure. In the bulk phase, this hierarchy HT2D/3D sample manifested obvious typical 3D perovskite (110) and (220) diffractions as well as quasi-2D diffractions at *q*_*z*_ from 0.32 to 0.75 Å^−1^, attributing to small-*n* quasi-2D phases (*n* = 2, 3, 4,…) [31, 32]. Moreover, at the small grazing-incident angle, the 3D perovskite scattering intensities were significantly attenuated, whereas the layered diffraction displayed a significantly sharp peak at *q*_*z*_ ~ 0.45 Å^−1^ with a narrow full width at half maxima, indicating a larger average grain size and higher ordered crystallite orientation caused by the quasi-2D perovskite phase on the top surface layer [28]. Thus, we inferred that small-*n* quasi-2D phases (*n* = 2, 3, 4,…) could be achieved by the following reaction:

(2) $$\begin{array}{c} n\text{MAPb}{\text{I}}_{3}+2\left(\text{TA}-\text{PMA}\right)\text{Br}⟶{\left(\text{TA}-\text{PMA}\right)}_{2}{\text{MA}}_{n-1}{\text{Pb}}_{n}{\text{I}}_{3n-1}{\text{Br}}_{2}+\text{MAI} \end{array}$$

The resultant MAI could be dissolved in IPA and further disappeared during spin-coating and thermal annealing process [33]. In the GIWAXS patterns of triple-cation perovskites (CsFAMA3D), it could also be clearly detected that pristine CsFAMA3D (Figures 2(i) and 2(j)) changed to a hierarchy HT2D/CsFAMA3D (Figures 2(k) and 2(l)) structure after the TA-PMA posttreatment.

### 2.4. Hole-Transporting Properties of HT2D Perovskite

In order to further investigate the hole mobility of HT2D perovskite, space-charge-limited-current (SCLC) measurements (Figure S6) based on hole-only devices were prepared as the following architecture: ITO/PEDOT:PSS/TA-PMA or HT2D/MoO_3_/Ag. The experimental hole mobility was significantly increased in more than one order of magnitude from 2.1 × 10^−4^ cm^2^ V^−1^ S^−1^ of TA-PMA to 2.6 × 10^−3^ cm^2^ V^−1^ S^−1^ of HT2D, which was comparable to doped Spiro-OMeTAD as HTL [34]. This enhanced hole mobility might be due to the following three reasons: (i) generally, the charge mobility of the 2D perovskite was relatively low owing to the insulating bulky organic layers which had a low hole mobility. In this work, the introduction of the triarylamine (TA) group could improve the hole mobility of the bulky organic molecules. Therefore, the TA-PMA-treated HT2D perovskite exhibited a high hole mobility. (ii) In addition, according to the DFT calculations, the TA units displayed a highly orientated arrangement along with the octahedral [PbI_6_]^4−^ framework in HT2D perovskite, since the PMA cation groups in TA-PMA could fully embed into the A-site at the (010) lattice plane of ABX_3_ 3D perovskite, whereas the molecule arrangement in the neat TA-PMA film was randomly unordered owing to the lack of perovskite framework as the template. (iii) Furthermore, the azimuth angle integration curve of HT2D diffraction peaks at *q* ~ 0.45 Å^−1^ displayed the appearance of this peak which was along the out-of-plane direction (*q*_*z*_) and not the in-plane direction (*q*_*xy*_), supporting the vertical packing of HT2D grown on substrate. These vertical packing HT2D can form a hole-transporting channel and thus perform higher hole mobility.

### 2.5. Morphology and Photoelectric Properties of TA-PMA Posttreatment Perovskite Films

**Figure 3:**
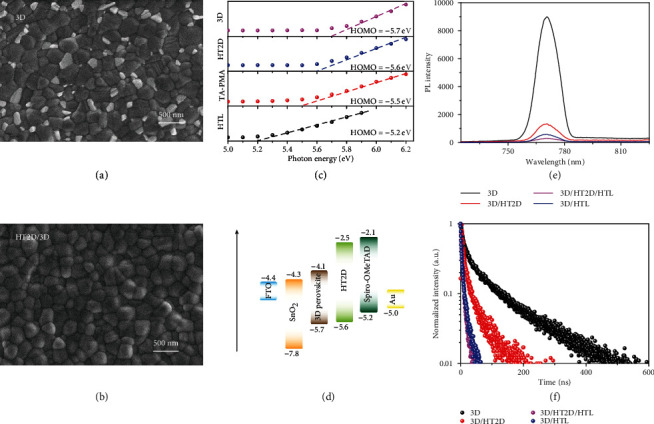
Morphology and photoelectric properties of TA-PMA posttreatment perovskite films: (a, b) top-view SEM of 3D (a) and HT2D/3D (b) perovskite films; (c) PESA spectra of 3D, HT2D, TA-PMA, and Spiro-OMeTAD films; (d) schematic energy diagram of TA-PMA-posttreated PSCs, energy levels of FTO, SnO_2_, Spiro-OMeTAD, and Au according to reference [2]; (e, f) images showing PL (e) and TRPL (f) spectra of 3D, 3D/HT2D, 3D/HTL, and 3D/HT2D/HTL.

We further investigated the morphology and crystalline phase changes of CsFAMA3D perovskite films induced by TA-PMA posttreatment. To simplify, in the following discussion, CsFAMA3D is abbreviated as 3D. As shown in Figure 3(a), the top-view SEM images of controlled 3D perovskites showed that many small grains of unreacted PbI_2_ were distributed in the grain boundaries of perovskite polycrystals, due to the disordered growth in perovskite crystallinity during thermal annealing process [33–37]. Significantly, after TA-PMA posttreatment (Figure 3(b)), these small grains seemed to completely disappear and crystal grains were significantly enlarged, due to the process of the aforementioned reaction (equation (1) and equation (2)) and Ostwald ripening [38]. Moreover, root mean square (RMS) surface roughness estimated from AFM images (Figure S7) demonstrated that the surface of the perovskite film became smoother and more compact for TA-PMA-modified 3D perovskites, ascribing to the formation of the HT2D perovskite layer [39].

Besides morphology modification, the surface-HT2D structure on the bulk-3D perovskite could also affect its work function and the interfacial band alignment. We performed photoelectron spectroscopy in air (PESA) on a series of samples (Figure 3(c)), exhibiting HOMO levels at −5.7 eV for 3D perovskite, −5.6 eV for HT2D, −5.5 eV for TA-PMA, and−5.2 eV for Spiro-OMeTAD as HTL, respectively. As shown in Figure 3(d), these gradually raised energy levels of 3D/HT2D/HTL architecture could effectively improve hole extraction and suppress interfacial nonradiative recombination between light-harvesting 3D perovskite and HTL in typical configuration. Furthermore, both steady-state photoluminescence (PL) (Figure 3(e)) and time-resolved photoluminescence (TRPL) (Figure 3(f)) spectra presented an effective enhancement on exciton/charge carrier extraction by using an intermediate HT2D layer. Compared to the 3D perovskite, the 3D/HT2D film showed a normalized PL intensity of 29.9% and calculated average PL decay time (*τ*_avg_) of 45.16 ns, which should be ascribed to the hole-extracting effect of the HT2D capping layer. Furthermore, these values of 3D/HT2D/HTL film could further reduce to 7.9% and 7.15 ns, which indicated that the gradual energy levels were more advantage for hole extraction compared to traditional 3D/HTL interface (13.8% and 9.61 ns). The fitted values of *τ*_1_, *τ*_2_, *A*_1_, and *A*_2_ and the calculated *τ*_avg_ of the samples are summarized in Table S1.

### 2.6. Photovoltaic Performance and Stability

**Figure 4:**
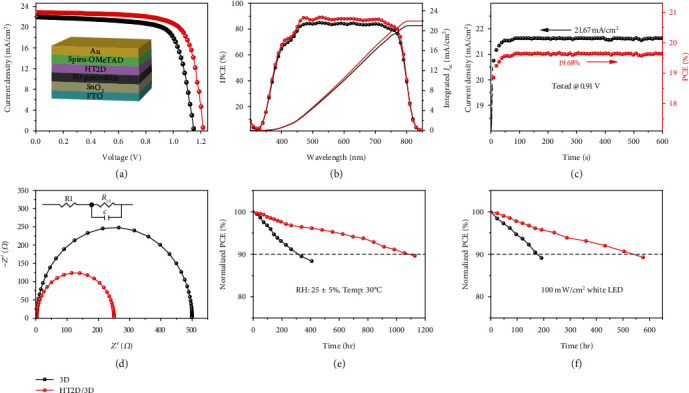
Device performance for 3D or HT2D/3D-based PSCs: (a) conventional n-i-p PSC architecture used in this work and *J* − *V* curves of the champion PSCs measured under 1 sun AM 1.5G with a scan rate of 10 mV s^−1^ using a 0.1 cm^2^ mask; (b) IPCE and the corresponding integrated *J*_sc_ for the best performing PSCs; (c) the stable output of a champion HT2D/3D-based device at a maximum power point of 0.91 V; (d) Nyquist plot of PSCs at a potential bias of 0.5 V and frequency range from 1 to 10^5^ Hz, in the dark; (e) stability test of PSCs stored in ambient air with a relative humidity of 25 ± 5% at 30°C; (f) the photostability of PSCs under illumination of 100 mW/cm^2^ white LED light in N_2_ glove box.

The surface-2D/bulk-3D hierarchy perovskite films were further fabricated into PSCs with a conventional planar n-i-p configuration of FTO/SnO_2_/perovskite/Spiro-OMeTAD/Au.  Figure 4(a) listed the current density–voltage (*J*–*V*) curves of HT2D/3D- and 3D-based PSCs. Noticeably, the mixed cation PSCs based on HT2D/3D delivered a high PCE up to 20.71% compared to the pristine 3D PSCs (PCE = 18.53%), achieving a photovoltage (*V*_oc_) of 1.21 V, a current density (*J*_sc_) of 22.81 mA cm^−2^, and a fill factor (FF) of 75.08%. The photovoltaic parameters of HT2D/3D PSCs were significantly enhanced with the reducing defect density of the TA-PMA-treated perovskite layers. As shown in Figure S8, the trap filled limit voltage (*V*_TFL_) of 3D and HT2D/3D perovskite films were 1.87 V and 0.70 V, respectively. According to the above logarithmic *J* − *V* analysis, the calculated defect concentration *N*_defects_ of HT2D/3D perovskite films (7.96 × 10^15^ cm^−3^) was an order of magnitude lower than the 3D counterpart (7.52 × 10^16^ cm^−3^) [40]. The lower defect density of HT2D/3D perovskite films indicated in which fewer nonradiative recombination centers existed due to less miscellaneous phases. The PV parameters of the devices are listed in Table S2. Their typical *J*–*V* hysteresis curves are listed in Figure S9. The HT2D/3D-based PSCs showed not only enhanced PV performance but also higher reproducibility than the pristine 3D-based PSCs (Figure S10). HT2D/3D-type PSCs processed with different contents of TA-PMA were analyzed to demonstrate the effect of TA-PMA on device performance. As shown in Figure S11 and Table S3, TA-PMA-treated PSCs exhibited a synchronous enhancement of *V*_oc_, *J*_sc_, and FF by optimizing an appropriate thickness of the HT2D layer. Nevertheless, using a similar concentration of PMA and TA as counterparts, PMA-modified PSC showed a slight enhancement on device performance, because PMA possessed only a low-dimensional unit but no improvement on hole transportation. TA-treated PSC presented even poor performance since it could not form a 2D structure and thus insulated hole extraction from perovskite to HTL (Figure S12 and Table S4). Incident photon-to-electron conversion efficiency (IPCE) measurements were carried out to validate the current densities of devices (Figure 4(b)). All IPCE spectra showed the onset of around 820 nm (~1% IPCE) and exhibited quantum efficiency values of over 80% from 400 to 780 nm. The integrated short-circuit current density (*J*_sc_) calculated from the IPCE spectrum was 21.94 mA cm^−2^ for HT2D/3D PSC and 20.99 mA cm^−2^ for 3D PSC, which matched well (~5%) the *J*_sc_ measured under AM 1.5G illumination. Furthermore, the HT2D/3D PSC also delivered a stabilized photocurrent of 21.67 mA cm^−2^, corresponding to a stabilized efficiency of 19.68%, under a constant voltage bias near the maximum power point (0.91 V) (Figure 4(c)), which was very close to the *J*–*V* scan efficiency.

Electrochemical impedance spectroscopy (EIS) analyses of PSCs were carried out to further characterize the charge transfer in the full devices based on HT2D/CsFAMA3D perovskites (Figure 4(d)) [41–43]. The Nyquist plots were obtained in the dark with an applied bias voltage of 0.5 V, and the fitted parameters are summarized in Figure 4(d). As shown in the equivalent circuit, the internal series resistance (*R*_s_) was related to the sheet resistance of the electrodes. The *R*_s_ values of the HT2D/CSFAMA3D and pristine CsFAMA3D PSCs were similar at 23.1 *Ω* and 31.8 *Ω*, respectively. On the other side, the charge-transfer resistance (*R*_ct_) generally refers to the contact resistance at all the interfaces such as the electrode/transporting layer/perovskite interface, which is corresponding to the semicircle in the high-frequency region [44]. At the *V*_oc_ of 0.5 V, the *R*_ct_ value of HT2D/3D PSC at 195 *Ω* was less than half the *R*_ct_ value of pristine CsFAMA3D PSC at 489 *Ω*, due to its better interfacial contact and charge transportation [45, 46]. The Nyquist plots of HT2D/CsFAMA3D and pristine 3D PSCs from 0 V–0.5 V showed similar trends shown in Figure S13 and Table S5.

In addition to the enhanced PV performance, the HT2D/CsFAMA3D PSCs exhibit excellent stability. In the unencapsulated HT2D/CsFAMA3D PSCs, 91% of the initial PCE could remain and a small hysteresis between forward and reverse JV sweep existed when stored in ambient condition with RH 25 ± 5% at 30°C for 1000 h (Figure 4(e) and Table S6). Contact angle analysis suggested that the high moisture stability of the TA-PMA-treated PSCs was ascribed to the more hydrophobic surface of the 2D structure, suggested by larger contact angles (64.2°) than 3D perovskite (48.6°) when using water droplets (Figure S14) [47]. Furthermore, the HT2D/3D PSC showed better photostability and 91% of the initial PCE remained after continuous illumination with white LED light for ~500 h in dry N_2_ glove box, while the pristine 3D PSC was below 90% of initial PCE at 200 h (Figure 4(f)).

## 3. Discussion

In this work, we have designed and synthesized a novel hole transporting organic salt TA-PMA, which can induce a surface-2D/bulk-3D hierarchy perovskite structure in both MAPbI_3_ and mixed-cation perovskite. The resulted HT2D perovskite on the 3D-bulk film surface could regulate the interfacial band alignment, suppress the interfacial charge recombination, improve the perovskite crystallization and film morphology, and enhance the hole mobility and charge extraction ability. As a result, all the photovoltaic parameters of the HT2D/3D PSC device including *J*_sc_, *V*_oc_, and FF were significantly enhanced, and the champion PCE of 20.71% was achieved along with the higher reproducibility than pristine 3D-based PSCs. Besides efficiency, another critical factor for photovoltaics was environmental stability and photostability under operating conditions. The stress tests demonstrated that HT2D/3D PSCs were much stabler over long-term operation against moisture and light soaking in contrast to the pristine 3D PSCs, owing to the hydrophobic nature properties of surface-2D layer. This work will offer a pathway to not only overcome the stability challenges for bulk 3D perovskite but also inspire the molecular design of novel hole-transporting 2D perovskite in the future.

## 4. Materials and Methods

### 4.1. Material Preparation

4-(Tert-butoxycarbonyl)benzeneboronic acid and 4-bromo-N, N-bis (4-methoxyphenyl) aniline were purchased from Energy Chemical Corp. Formamidinium iodide (FAI), methylammonium bromine (MABr), and lead iodide (PbI_2_) were purchased from ZhongNeng Corp. Lead bromide (PbBr_2_), methanaminium iodide (MAI), and Spiro-OMeTAD were purchased from Xi'an Polymer Light Technology. Other materials were bis(trifluoromethane) sulfonimide lithium salt (99.95%, Aldrich), 4-tert-butylpyridine (99.9%, TCI), FK 209 Co (III) TFSI salt (Xi' an Polymer Light Technology Corp), and SnO_2_ colloidal solution (15% in H_2_O colloidal dispersion, Alfa Aesar).

### 4.2. Preparation of HT2D Perovskite Films

The neat HT2D perovskite film was prepared as follows: 0.1 mmol of TA-PMA and 0.05 mmol of PbI_2_ were dissolved in 1 mL of DMF, spin-coated on the substrate at 3000 rpm for 30 s, and then annealed at 100°C for 10 minutes.

### 4.3. Preparation of HT2D/3D Perovskite Films

The hierarchy HT2D/3D perovskite film was prepared as follows: TA-PMA was dissolved in IPA in 3 mg/mL, then spin-coated on the 3D perovskite film at 3000 rpm for 30 s. The posttreated perovskite film was annealed at 100°C for 10 min.

### 4.4. SCLC Mobility Measurements

The hole-only device for hole mobility measurements consisted of ITO/PEDOT:PSS/TA-PMA:PbI_2_ (or TA-PMA)/MoO_3_/Ag. A preetched ITO substrate was treated with UV–ozone for 30 min, and then a 40 nm PEDOT:PSS layer was spin-coated onto the ITO substrate from an aqueous solution, followed by annealing at 150°C for 10 min. 0.1 mmol of TA-PMA and 0.05 mmol of PbI_2_ (or neat 0.1 mmol of TA-PMA) were dissolved in 1 mL of DMF, spin-coated on the PEDOT:PSS layer at 3000 rpm for 30 s, and then annealed at 100°C for 10 minutes on a hot stage. A bilayer cathode structure of MoO_3_ (20 nm)/Ag (100 nm) was thermally evaporated on top of the TA-PMA:PbI_2_ (or TA-PMA) film. By using the following equation, the hole mobilities of the devices were determined with the SCLC method

(3) $$\begin{array}{c} J=\frac{9}{8}\varepsilon_{\text{r}}\varepsilon_{0}\mu_{\text{h}}\frac{V^{2}}{L^{3}}, \end{array}$$

where *ε*_0_ is the permittivity of free space, *ε*_r_ is the dielectric constant of the TA-PMA:PbI_2_, *μ*_h_ is the hole mobility, *V* is the voltage drop across the device, and *L* is the TA-PMA:PbI_2_ film thickness; in formula, *V* = *V*_appl_ − *V*_r_ − *V*_bi_, *V*_appl_ is the applied voltage to the device, *V*_r_ is the voltage drop due to constant resistance and series resistance across the electrodes, and *V*_bi_ is the built-in voltage due to the difference in work function of the two electrodes. The current density versus voltage characteristics were recorded on a Keithley 2400 source meter.

### 4.5. XRD Measurements

In order to clearly identify diffraction peaks, a simple component MAPbI_3_ perovskite was adopted for XRD analysis. 1 mmol MAI and 1 mmol PbI_2_ were dissolved in 800 *μ*L mixed solvent of DMF/DMSO (4 : 1, by volume) as a precursor solution. The MAPbI_3_ perovskite films were deposited by spun a 30 *μ*L precursor solution at 4000 rpm for 30 s with an acceleration speed of 1000 rpm. During this step, 100 *μ*L antisolvent of ethyl acetate (EA) was dropped at the last 5th second as the antisolvent. The films were annealed at 100°C for 20 min and waited until cooling to room temperature. Then, TA-PMA (or PMA, TA) dissolved in IPA was spin-coated on the MAPbI_3_ perovskite film at 3000 rpm for 30 s; the posttreated perovskite film was annealed at 100°C for 10 min. The different perovskite films were tested by an X-ray diffractometer (XRD, D8 Advance) using CuK*α* radiation.

### 4.6. PSC Device Fabrication of Mixed Cation Perovskite

FTO glass substrates (Nippon Sheet Glass) were ultrasonically cleaned by glass detergent (1 vol% in deionized water), deionized water, acetone, and ethanol for 15 min, in sequence. Substrates were treated with UV–ozone for 30 min to remove the last traces of organic residues. For the SnO_2_ films, the as-purchased SnO_2_ colloidal dispersion was diluted by water (1 : 3 wt) and then spin-coated onto the clean FTO glass substrates at 3000 rpm for 30 s and repeated for two times, then annealed at 150°C for 30 min. The mixed CsFAMA perovskite layers were deposited by spinning a 30 *μ*L CsFAMA perovskite solution (see preparation details as follows) in a two-step procedure at 6000 and 6000 rpm for 30 and 10 s with an acceleration speed of 1000 and 6000 rpm. During the first step, 100 *μ*L antisolvents of EA were dropped at the last 5th second, respectively. The films were annealed at 100°C for 60 min and waited until cooling to room temperature. Then, 3 mg TA-PMA/mL IPA solution was spin-coated onto the 3D perovskite film at 3000 rpm for 30s; the posttreated perovskite film was annealed at 100°C for 10 min. A 35 *μ*L Spiro-OMeTAD solution (see preparation details as follows) was spun on the corresponding mixed perovskite films at 3000 rpm for 30 s. Finally, 80 nm of gold was evaporated as the back electrode to form the whole devices.

### 4.7. Preparation of the Mixed Perovskite Precursor Solution

“(FA_0.85_MA_0.15_) Pb(I_0.85_Br_0.15_)_3_” was a perovskite formulation, written as “FAMA.” The FAMA-mixed perovskite precursor solution was prepared by dissolving PbI_2_ (1.218 M), PbBr_2_ (0.182 M), FAI (1.218 M), and MABr (0.182 M) in a mixed solvent of DMF/DMSO (4 : 1, by volume). 30 *μ*L CsI solution (predissolved as a 1.5 M stock solution in DMSO) was added to the FAMA-mixed perovskite precursor to achieve the desired triple Cs_0.05_FA_0.81_MA_0.14_PbI_2.55_Br_0.45_ perovskite solution (labeled as CsFAMA).

### 4.8. Measurement and Calculation of *N*_defects_

We have measured and calculated the *N*_defects_ according to the reference [8]. *J* − *V* characteristics of devices (ITO/perovskite/Au), used for estimating the SCLC defect concentration (*N*_defects_ = 2 *ɛɛ*_0_*V*_TFL_/*eL*^2^), *V*_TFL_ is the trap-lied limit voltage, *ɛ* and *ɛ*_0_ are the dielectric constants of perovskite and vacuum permittivity, *L* is the thickness of the perovskite film, and *e* is the elementary charge.

### 4.9. Instrument Details

The NMR spectra were obtained on a Bruker 400 MHz instrument. The different perovskite films were tested by an X-ray diffractometer (XRD, D8 Advance) using CuK*α* radiation; the optical absorption of the perovskite samples was measured using a UV–vis spectrophotometer (PerkinElmer Lambda 750). The PESA (photoelectron spectroscopy in air) was measured with Nanjing Sunny Tech's IPS-4 Ionization Energy Measurement System. The surface morphologies and microstructures of the perovskite films were investigated using a field-emission scanning electron microscopy (FESEM, Zeiss Ultra Plus). The steady-state photoluminescence (PL) spectra were obtained using a PL microscopic spectrometer (Flex One, Zolix, China). The time-resolved photoluminescence (TRPL) was measured at 780 nm using excitation with a 510 nm light pulse from Delta Flex Fluorescence Lifetime System (Horiba Scientific Com., Japan). GIWAXS experiments were performed at BL14B1 and BL16B1 beamlines at Shanghai Synchrotron Radiation Facility. The photocurrent density-voltage curves of the perovskite solar cells were measured using a solar simulator (Oriel 94023A, 300 W) and a Keithley 2400 source meter. The intensity (100 mW/cm^2^) was calibrated using a standard Si solar cell (Oriel, VLSI standards). All the devices were tested under AM 1.5G sunlight (100 mW/cm^2^) using a metal mask of 0.1 cm^2^ with a scan rate of 10 mV/s. IPCE measurements were carried out in DC mode by using a Keithley 2400 source meter and a SOFN 7ISW752 monochromator equipped with a 500 W Xenon lamp. The wavelength sampling interval was 5 nm, and the current sampling time was 1 s, which was fully controlled by a computer. A Hamamatsu S1337-1010BQ silicon diode used for IPCE measurements was calibrated at the National Institute of Metrology, China. The EIS measurements were carried out by an EC-lab (SP300).

## Data Availability

The data that support the finding of this study are available from the corresponding author upon reasonable request.

## Supplementary Materials

Supplementary Materials Figure S1: synthetic routes of TA-PMA. Molecular structures of PMA, TA, and TA-PMA. Figure S2: *^1^H*-NMR of tert-butyl((4′-(bis(4-methoxyphenyl)amino)-[1,1′-biphenyl]-4-yl)methyl)carbamate (1). Figure S3: *^1^H*-NMR of (4′-(bis(4-methoxyphenyl)amino)-[1,1′-biphenyl]-4-yl)methan-aminium bromide (TA-PMA). Figure S4: N-N atom distances of PMA units (~6.302 Å), TA units (~6.401 Å), and MA units (~6.435 Å) calculated by DFT. Figure S5: density of state of TA-PMA and MAPbI3 calculated by DFT. Figure S6: space-charge-limited-current (SCLC) curves of TA-PMA or TA-PMA: PbI_2_. Figure S7: AFM topography images and RMS surface roughness of 3D (a) and HT2D/3D (b) perovskite films. Figure S8: dark *J* − *V* characteristic curves of 3D and HT2D/3D. Figure S9: photovoltaic performances of champion 3D and HT2D/3D PSCs measured under forward and reverse scan direction with scan rate 10 mV s^−1^. Figure S10: efficiency distribution histograms of 30 independent PSCs based on 3D or HT2D/3D. Figure S11: *J* − *V* curves of HT2D/3D-based PSCs using different TA-PMA concentrations. Figure S12: *J* − *V* curves of the champion PSCs based on perovskites modified by TA-PMA, phenylmethylammonium (PMA) bromide, and dimethoxy-triarylamine (TA) at the concentration of 3 mg/mL used 3D perovskite as the counterpart. Figure S13: Nyquist plot of (a) pristine 3D and (b) HT2D/3D PSCs at different potential biases from 0 V to 0.5 V and frequency range from 1 to 10^5^ Hz, in the dark. Figure S14: the contact angles of deionized water droplets on the (a) 3D and (b) HT2D/3D perovskite films. Table S1: parameters of the time-resolved photoluminescence (TRPL) spectroscopy on the glass. Table S2: photovoltaic performance of the best PSCs based on 3D or HT2D/3D under the 100 mW cm^−2^ illumination. Table S3: the PV parameters of HT2D/3D-based PSCs in different TA-PMA concentrations. Table S4: the PV parameters of perovskite-modified TA-PMA, PMA, and TA used pristine 3D perovskite as the counterpart, under the 100 mW cm^−2^ illumination. Table S5: fitted values of different electronic parameters from dark Nyquist plots of 3D and HT2D/3D with voltage from 0 V to 0.5 V. Table S6: PV parameters of HT2D/3D in the stability test.
