# Supplementary material for: Hole-Transporting Low-Dimensional Perovskite for Enhancing Photovoltaic Performance
Source: Research (Wash D C). 2021 May 28;2021:9797053. doi: 10.34133/2021/9797053 (PMC8328399; doi:10.34133/2021/9797053)
Supplement: Supplementary Materials — Figure S1: synthetic routes of TA-PMA. Molecular structures of PMA, TA, and TA-PMA. Figure S2: 1H-NMR of tert-butyl((4′-(bis(4-methoxyphenyl)amino)-[1,1′-biphenyl]-4-yl)methyl)carbamate (1). Figure S3: 1H-NMR of (4′-(bis(4-methoxyphenyl)amino)-[1,1′-biphenyl]-4-yl)methan-aminium bromide (TA-PMA). Figure S4: N-N atom distances of PMA units (~6.302 Å), TA units (~6.401 Å), and MA units (~6.435 Å) calculated by DFT. Figure S5: density of state of TA-PMA and MAPbI3 calculated by DFT. Figure S6: space-charge-limited-current (SCLC) curves of TA-PMA or TA-PMA: PbI2. Figure S7: AFM topography images and RMS surface roughness of 3D (a) and HT2D/3D (b) perovskite films. Figure S8: dark J − V characteristic curves of 3D and HT2D/3D. Figure S9: photovoltaic performances of champion 3D and HT2D/3D PSCs measured under forward and reverse scan direction with scan rate 10 mV s−1. Figure S10: efficiency distribution histograms of 30 independent PSCs based on 3D or HT2D/3D. Figure S11: J − V curves of HT2D/3D-based PSCs using different TA-PMA concentrations. Figure S12: J − V curves of the champion PSCs based on perovskites modified by TA-PMA, phenylmethylammonium (PMA) bromide, and dimethoxy-triarylamine (TA) at the concentration of 3 mg/mL used 3D perovskite as the counterpart. Figure S13: Nyquist plot of (a) pristine 3D and (b) HT2D/3D PSCs at different potential biases from 0 V to 0.5 V and frequency range from 1 to 105 Hz, in the dark. Figure S14: the contact angles of deionized water droplets on the (a) 3D and (b) HT2D/3D perovskite films. Table S1: parameters of the time-resolved photoluminescence (TRPL) spectroscopy on the glass. Table S2: photovoltaic performance of the best PSCs based on 3D or HT2D/3D under the 100 mW cm−2 illumination. Table S3: the PV parameters of HT2D/3D-based PSCs in different TA-PMA concentrations. Table S4: the PV parameters of perovskite-modified TA-PMA, PMA, and TA used pristine 3D perovskite as the counterpart, under the 100 mW cm−2 illumination. [file 9797053.f1.docx]

Hole-Transporting Low-Dimensional Perovskite for Enhancing Photovoltaic Performance

Fangfang Wang^1^, Qing Chang^1^, Yikai Yun^1^, Sizhou Liu^1^, You Liu^1^, Jungan Wang^1^, Yinyu Fang^1^, Zhengchun Cheng^1^, Shanglei Feng^3^, Lifeng Yang^3^, Yingguo Yang^3^*, Wei Huang^2^*, Tianshi Qin^1^*

^1^ Key Laboratory of Flexible Electronics (KLOFE) & Institute of Advanced Materials (IAM), Nanjing Tech University (NanjingTech), 30 South Puzhu Road, Nanjing 211816, China.

^2^ Frontiers Science Center for Flexible Electronics (FSCFE) & Shaanxi Institute of Flexible Electronics (SIFE), Northwestern Polytechnical University (NPU), 127 West Youyi Road, Xi'an 710072, China

^3^ Shanghai Synchrotron Radiation Facility (SSRF), Shanghai Advanced Research Institute, Shanghai Institute of Applied Physics, Chinese Academy of Sciences, 239 Zhangheng Road, Shanghai 201204, P. R. China.

Correspondence should be addressed to Prof. Tianshi Qin; [iamtsqin@njtech.edu.cn](mailto:iamtsqin@njtech.edu.cn) Prof. Wei Huang; [iamwhuang@nwpu.edu.cn](mailto:iamwhuang@nwpu.edu.cn) and Prof. Yingguo Yang. [yangyingguo@sinap.ac.cn](mailto:yangyingguo@sinap.ac.cn)

**Figure S1 Synthetic routes of TA-PMA. Molecular structures of PMA, TA and TA-PMA.**


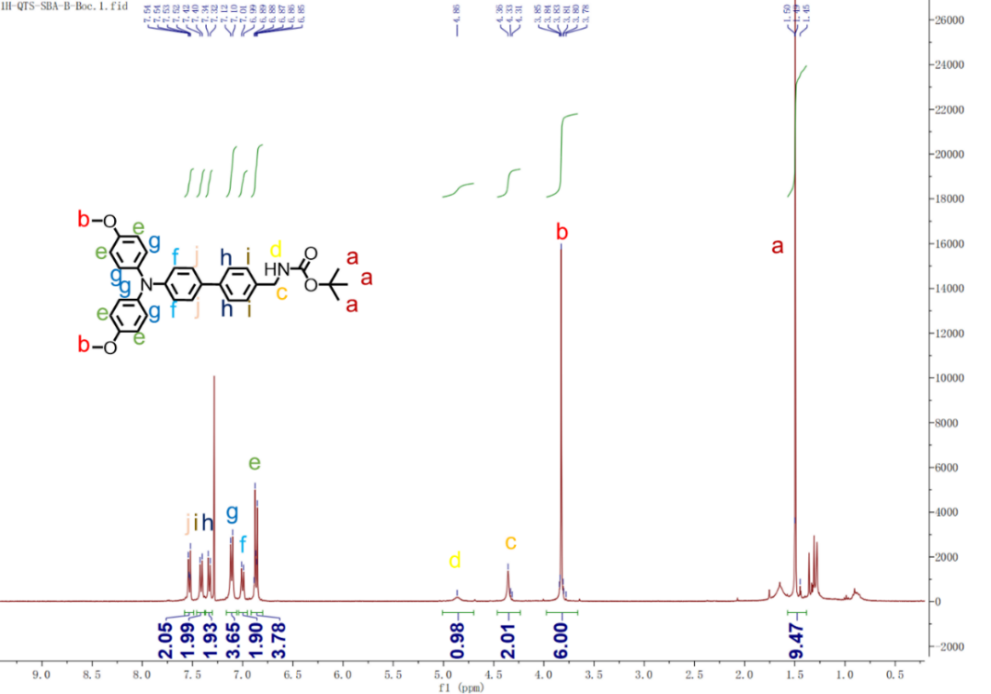


**Figure S2 1H-NMR of tert-butyl((4'-(bis(4-methoxyphenyl)amino)-[1,1'-biphenyl]-4-yl)methyl)carbamate (1).**


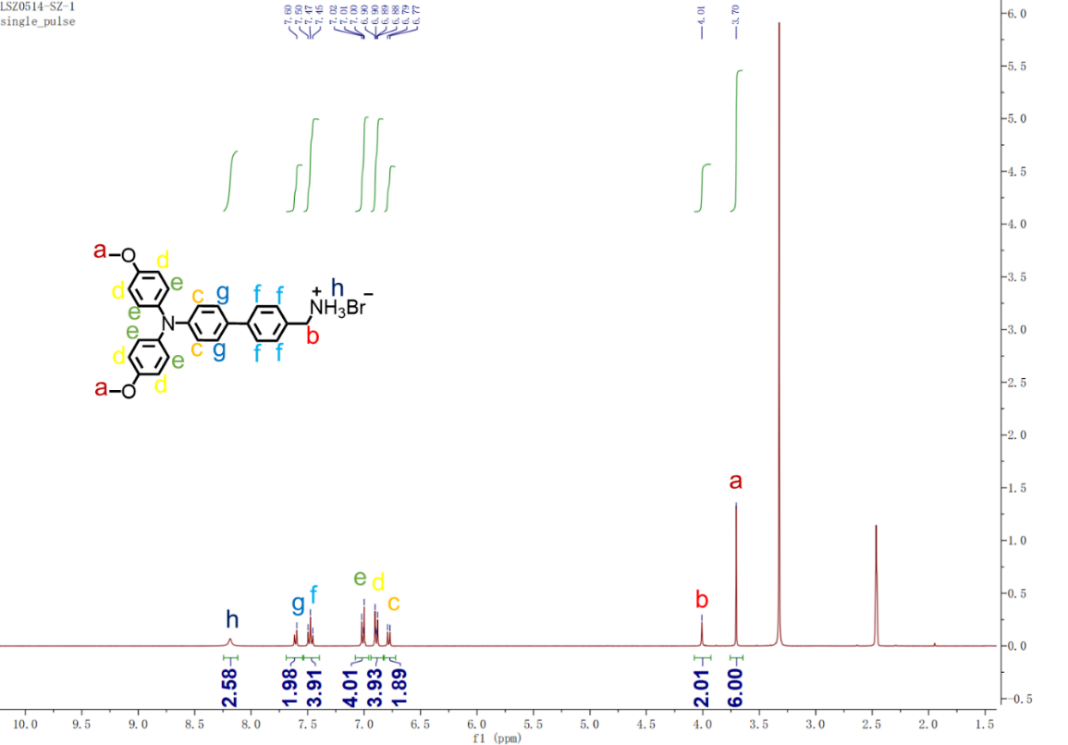


**Figure S3 ^1^H-NMR of (4'-(bis(4-methoxyphenyl)amino)-[1,1'-biphenyl]-4-yl)methan-aminium bromide (TA-PMA).**


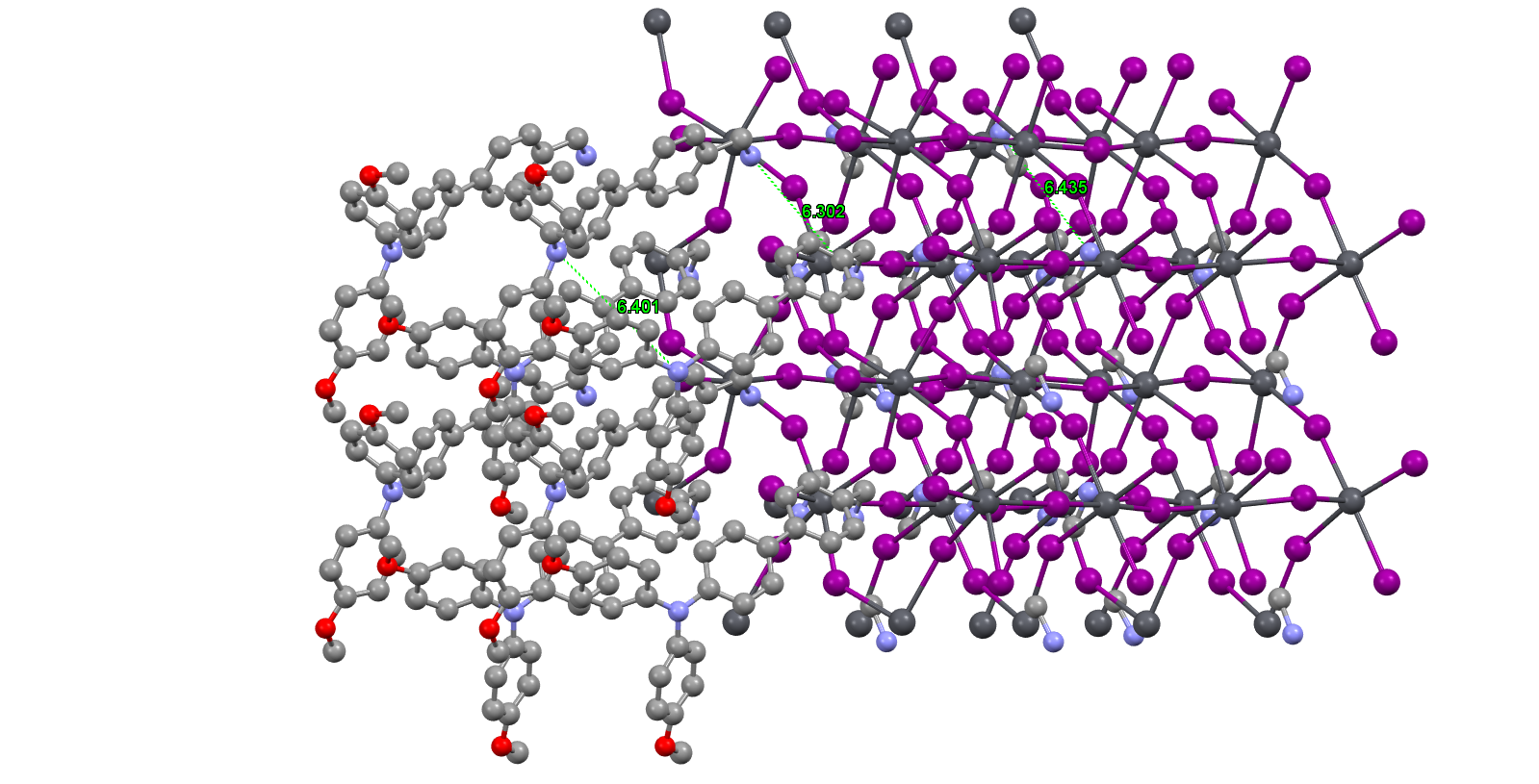


**Figure S4 N-N atom distances of PMA units (~6.302 Å), TA units (~6.401 Å), and MA units (~6.435 Å) calculated by DFT.**

**Figure S5** **Density of state of TA-PMA and MAPbI_3_ calculated by DFT.**

**
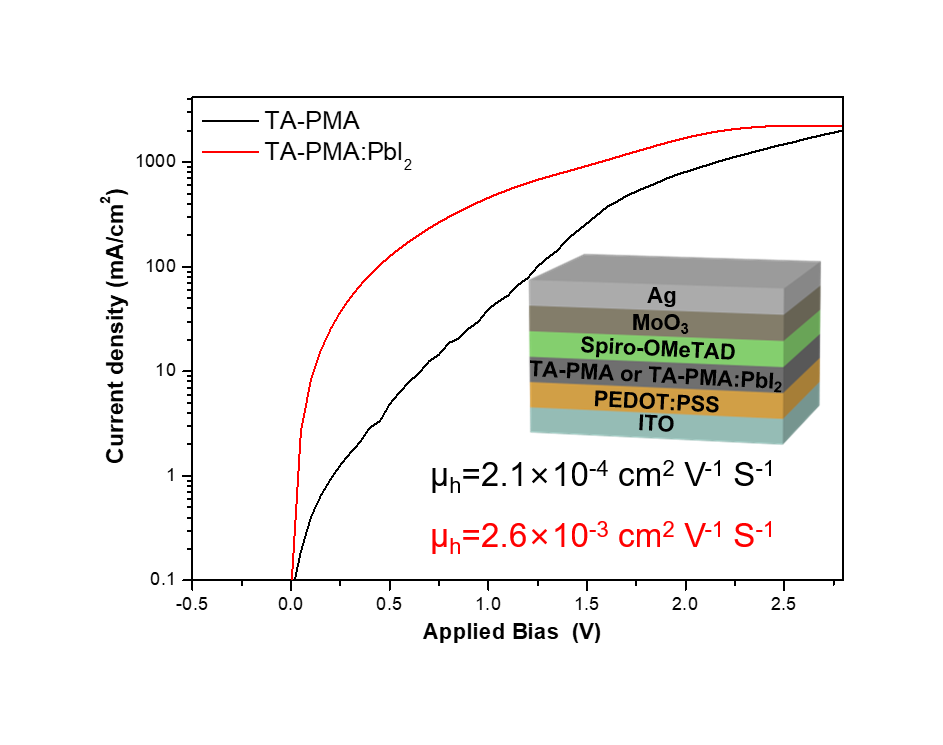
**

**Figure S6 Space-charge-limited-current (SCLC) curves of TA-PMA or TA-PMA: PbI_2_.**

**
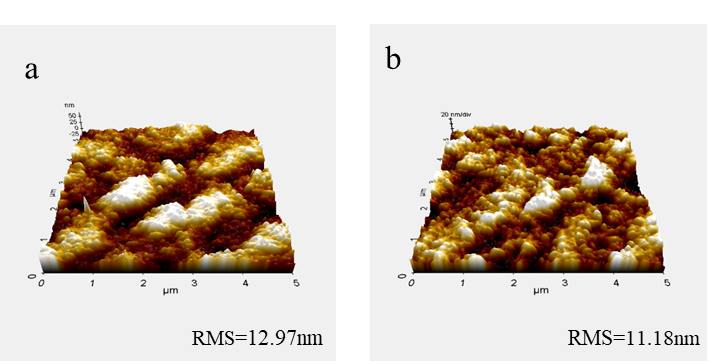
**

**Figure S7 AFM topography images and RMS surface roughness of 3D (a) and HT2D/3D (b) perovskite films.**

**

**

**Figure S8 Dark J−V characteristic curves of 3D and HT2D/3D.**

**

**

**Figure S9 Photovoltaic performances of champion 3D and HT2D/3D PSCs measured under forward and reverse scan direction with scan rate 10 mV s^-1^.**

**

**

**Figure S10 Efficiency distribution histograms of 30 independent PSCs based on 3D or HT2D/3D.**


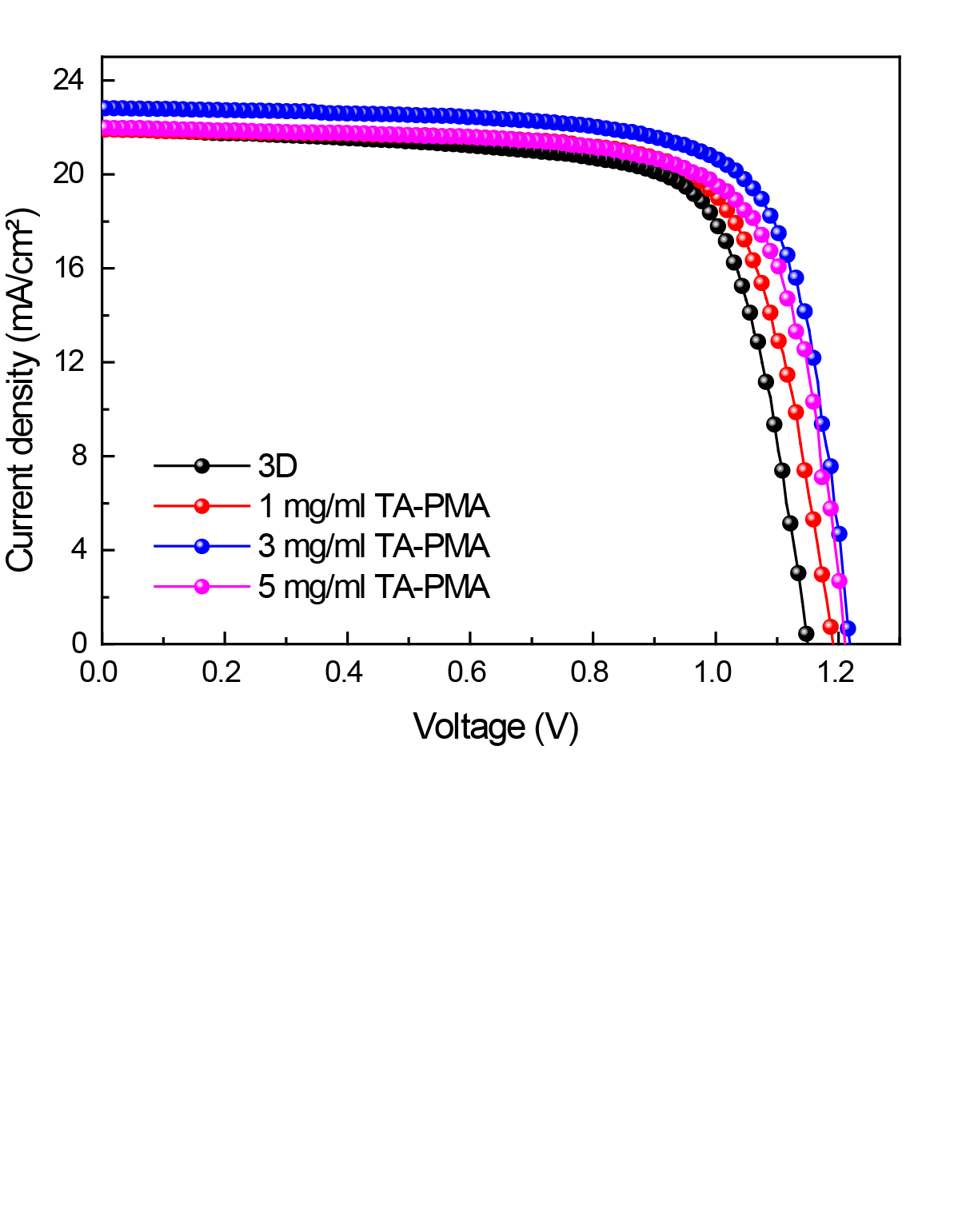


**Figure S11 J−V curves of HT2D/3D based PSCs using different TA-PMA concentrations.**

hysteresis
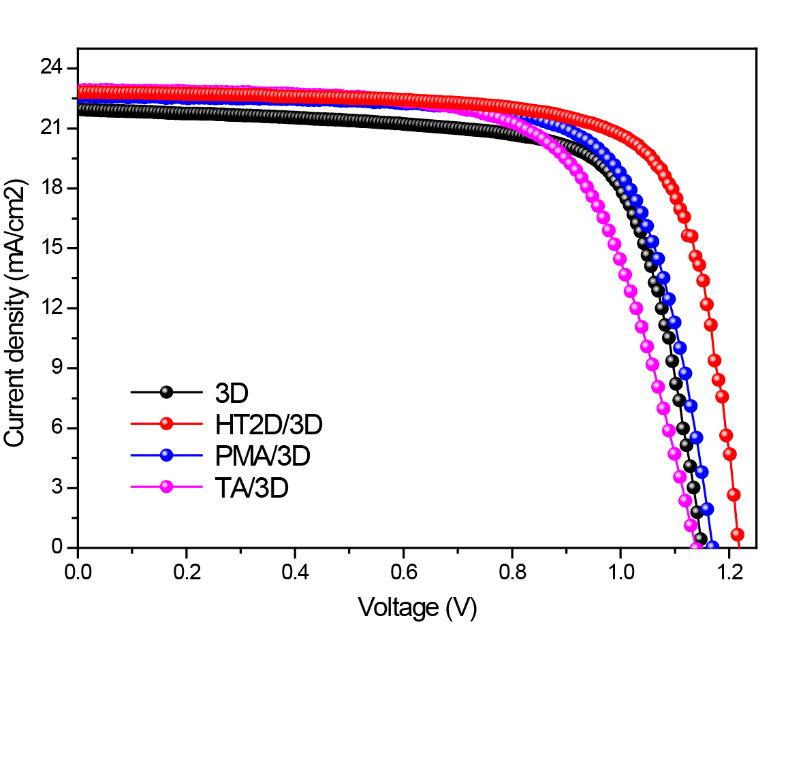


**Figure S12 J−V curves of the champion PSCs based on perovskites modified by TA-PMA, phenylmethylammonium (PMA) bromide and dimethoxy-triarylamine (TA) at the concentration of 3mg/mL used 3D perovskite as the counterpart.**


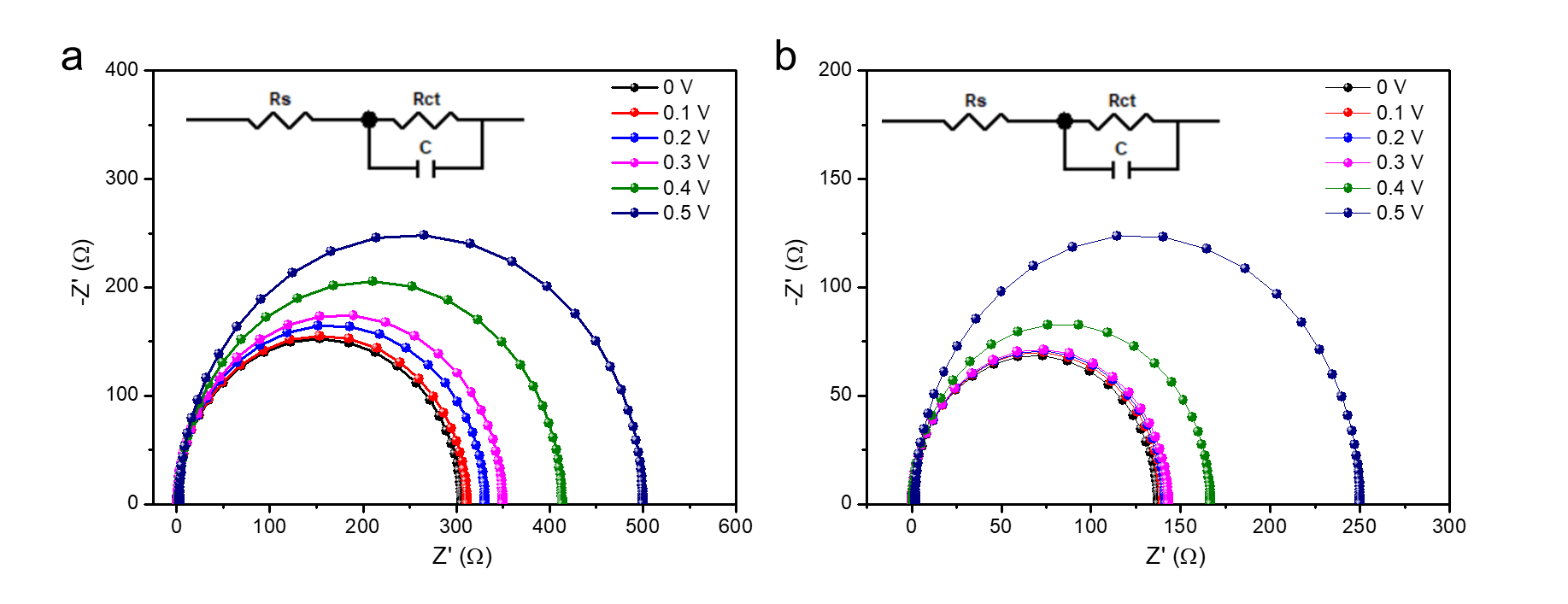


**Figure S13 Nyquist plot of (a) pristine 3D and (b) HT2D/3D PSCs at different potential bias from 0 V to 0.5 V, and frequency range from 1 to 10^5^ Hz, in the dark.**

**
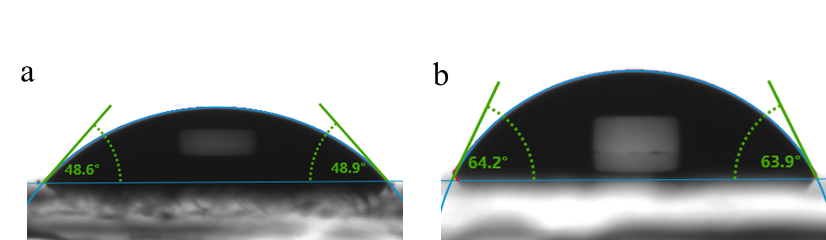
**

**Figure S14 The contact angles of deionized water droplets on the (a) 3D and (b) HT2D/ 3D perovskite films.**

**Table. S1 Parameters of the time-resolved photoluminescence (TRPL) spectroscopy on the glass.**

| Devices | τ_1_ [ns] | A_1_ [%] | τ_2_ [ns] | A_2_ [%] | τavg[ns] |
| --- | --- | --- | --- | --- | --- |
| pristine | 51.96 | 40.30 | 176.95 | 59.70 | 91.63 |
| with HT2D | 21.10 | 40.54 | 86.23 | 59.46 | 45.16 |
| pristine/HTL | 4.15 | 58.42 | 26.85 | 41.58 | 9.61 |
| with HT2D/HTL | 3.70 | 56.77 | 19.47 | 43.23 | 7.15 |

The PL decay time and amplitudes are modeled using a biexponential expression:

$f\left( t \right)=\sum_{i} A_{i}\exp\left( -\frac{t}{\tau_{i}} \right)+K$ (1)

where *A*_i_ is the decay amplitude, *τ*_i_ is the decay time and *K* is a constant for the base-line offset. The average PL decay times (τ_avg_) are further estimated using the τi and A_i_ values from the fitted curve data Table S1 using:

$\tau_{ave}=\frac{\sum A_{i}\tau_{i}^{2}}{\sum A_{i}\tau_{i}}$ (2)

**Table. S2 Photovoltaic performance of the best PSCs based on 3D or HT2D/3D under the 100 mW cm-2 illumination.**

| Devices | Scan direction | *V*_oc_[V] | *J*_sc_[mA/cm^2^] | FF[%] | PCE [%] |
| --- | --- | --- | --- | --- | --- |
| pristine | RS | 1.15 | 21.95 | 73.41 | 18.53 |
|  | FS | 1.15 | 21.86 | 70.42 | 17.70 |
| with HT2D | RS | 1.21 | 22.81 | 75.08 | 20.71 |
|  | FS | 1.20 | 22.49 | 74.72 | 20.16 |

**Table. S3 The PV parameters of HT2D/3D based PSCs in different TA-PMA concentrations.**

| Devices | *V*_oc_[V] | *J*_sc_[mA/cm^2^] | FF[%] | PCE [%] |
| --- | --- | --- | --- | --- |
| None (pure 3D) | 1.15 | 21.95 | 73.41 | 18.53 |
| 1mg/mL TA-PMA | 1.19 | 21.90 | 73.88 | 19.26 |
| 3mg/mL TA-PMA | 1.21 | 22.81 | 75.08 | 20.71 |
| 5mg/mL TA-PMA | 1.21 | 21.99 | 73.52 | 19.59 |

.

**Table. S4 The PV parameters of perovskites modified TA-PMA, PMA and TA used pristine 3D perovskite as the couterpart, under the 100 mW cm-2 illumination.**

| Devices | *V*_oc_[*V*] | *J*_sc_[mA/cm^2^] | FF[%] | PCE [%] |
| --- | --- | --- | --- | --- |
| pristine | 1.15 | 21.95 | 73.41 | 18.53 |
| 3mg/mL TA-PMA | 1.21 | 22.81 | 75.08 | 20.71 |
| 3mg/mL PMA | 1.17 | 22.58 | 72.59 | 19.18 |
| 3mg/mL TA | 1.14 | 22.91 | 67.41 | 17.59 |

**Table. S5 Fitted values of different electronic parameters from dark Nyquist plots of 3D and HT2D/3D with voltage from 0 V to 0.5 V.**

| **Voltage (V)** | 3D | | HT2D/3D | |
| --- | --- | --- | --- | --- |
|  | *R*_s_ | *R*_ct_ | *R*_s_ | *R*_ct_ |
| **0** | 37.01 | 304.70 | 23.94 | 137.09 |
| **0.1** | 38.43 | 310.56 | 22.90 | 139.96 |
| **0.2** | 40.62 | 330.12 | 22.07 | 141.36 |
| **0.3** | 46.94 | 348.76 | 27.47 | 142.83 |
| **0.4** | 123.10 | 410.92 | 52.66 | 166.12 |
| **0.5** | 278.9 | 496.10 | 199.40 | 248.07 |

**Table S6 PV parameters of HT2D/3D in the stability test.**

| **Devices** | Scan direction | *V*_oc_[V] | *J*_sc_[mA/cm^2^] | FF[%] | PCE [%] | HI |
| --- | --- | --- | --- | --- | --- | --- |
| **0 h** | RS | 1.21 | 22.81 | 75.08 | 20.71 | 0.02656 |
|  | FS | 1.20 | 22.49 | 74.72 | 20.16 |  |
| **176 h** | RS | 1.20 | 22.75 | 74.14 | 20.24 | 0.02816 |
|  | FS | 1.20 | 22.32 | 73.44 | 19.67 |  |
| **352 h** | RS | 1.20 | 22.51 | 73.85 | 19.95 | 0.02857 |
|  | FS | 1.19 | 22.11 | 73.65 | 19.38 |  |
| **528 h** | RS | 1.20 | 22.16 | 74.27 | 19.75 | 0.03240 |
|  | FS | 1.19 | 21.89 | 73.36 | 19.11 |  |
| **704 h** | RS | 1.19 | 22.21 | 73.74 | 19.49 | 0.03642 |
|  | FS | 1.18 | 21.87 | 72.77 | 18.78 |  |
| **880 h** | RS | 1.18 | 21.79 | 73.27 | 18.84 | 0.04193 |
|  | FS | 1.18 | 21.43 | 71.38 | 18.05 |  |
| **1056 h** | RS | 1.18 | 21.56 | 73.46 | 18.69 | 0.04441 |
|  | FS | 1.18 | 21.22 | 71.33 | 17.86 |  |

**Computational Details.** Density function theory calculation were performed by using the CP2K package. (*1*) PBE functional (*2*) with Grimme D3 correction (*3*) was used to describe the systemm. Kohn-Sham DFT has been used as the electronic structure method in the framework of the Gaussian and plane waves method. (*4, 5*) The Goedecker-Teter-Hutter (GTH) pseudopotentials, (*6, 7*) DZVP- MOLOPT-GTH basis sets (*4*) were utilized to describe the molecules. A plane-wave energy cut-off of 500 Ry has been employed. The PSK is modelled by a four atomic layer p(4×4) <010> surface. To maintain the bulk properties, the bottom two atomic layers are kept fixed during the optimizations.

**Reference.**

[1] Jürg Hutter, Marcella Iannuzzi, Florian Schiffmann, and Joost Vande Vondele. Cp2k: atomistic simulations of condensed matter systems. Wiley Interdisciplinary Reviews: Computational Molecular Science, 4(1):15–25, 2014.

[2] John P Perdew, Kieron Burke, and Matthias Ernzerhof. Generalized gradient approximation made simple. Physical review letters, 77(18):3865, 1996.

[3] Stefan Grimme. Semiempirical gga-type density functional constructed with a long-range dis- persion correction. Journal of computational chemistry, 27(15):1787–1799, 2006.

[4] Joost Vande Vondele and Juerg Hutter. Gaussian basis sets for accurate calculations on molec- ular systems in gas and condensed phases. The Journal of chemical physics, 127(11):114105, 2007.

[5] Joost Vande Vondele, Matthias Krack, Fawzi Mohamed, Michele Parrinello, Thomas Chassaing, and Jürg Hutter. Quickstep: Fast and accurate density functional calculations using a mixed gaussian and plane waves approach. Computer Physics Communications, 167(2):103–128, 2005.

[6] S Goedecker, M Teter, and Jürg Hutter. Separable dual-space gaussian pseudopotentials. Physical Review B, 54(3):1703, 1996.

[7] C Hartwigsen, Sephen Gœdecker, and Jürg Hutter. Relativistic separable dual-space gaussian pseudopotentials from h to rn. Physical Review B, 58(7):3641, 1998.
